# Education First: Promoting LGBT+ Friendly Healthcare with a Competency-Based Course and Game-Based Teaching

**DOI:** 10.3390/ijerph17010107

**Published:** 2019-12-22

**Authors:** Hsing-Chen Yang

**Affiliations:** Graduate Institute of Gender Studies, Kaohsiung Medical University, Kaohsiung 80708, Taiwan; yhckmu@gmail.com

**Keywords:** assessment as learning, medical student, game-based teaching, gender competency, LGBT+ friendly healthcare

## Abstract

How, apart from by conveying professional knowledge, can university medical education nurture and improve the gender competency of medical students and thereby create an LGBT+ friendly healthcare environment? This study explored the use of game-based teaching activities in competency-based teaching from the perspective of competency-based medical education (CBME) and employed a qualitative case-study methodology. We designed an LGBT+ Health and Medical Care course in a medical school. Feedback was collected from two teachers and 19 medical students using in-depth interviews and thematic analysis was used to analyze the collected data. The findings of this study were as follows: (1) Games encouraged student participation and benefited gender knowledge transmission and transformation through competency learning, and (2) games embodied the idea of assessment as learning. The enjoyable feeling of pressure from playing games motivated students to learn. Using games as both a teaching activity and an assessment tool provided the assessment and instant feedback required in the CBME learning process. Game-based teaching successfully guided medical students to learn about gender and achieve the learning goals of integrating knowledge, attitudes, and skills. To fully implement CBME using games as teaching methods, teaching activities, learning tasks, and assessment tools, teachers must improve their teaching competency. This study revealed that leading discussions and designing curricula are key in the implementation of gender competency-based education; in particular, the ability to lead discussions is the core factor. Game-based gender competency education for medical students can be facilitated with discussions that reinforce learning outcomes to achieve the objectives of gender equality education and LGBT+ friendly healthcare. The results of this study indicated that game-based CBME with specific teaching strategies was an effective method of nurturing the gender competency of medical students. The consequent integration of gender competency into medical education could achieve the goal of LGBT+ friendly healthcare.

## 1. Introduction

Gender is a major source of concern in healthcare environments. A psychiatrist with 10 years of practical experience, including with numerous LGBT+ patients, contended that doctors are able to more easily help patients who are willing to fully reveal their true selves and share their feelings [1]. However, the extent to which LGBT+ patients feel comfortable revealing themselves and the additional help that doctors can provide depend on numerous conditions and factors; of these, doctors’ gender competency is a key factor.

In many places, good health is considered to be a basic human right to which everyone is entitled. From the perspective of gender equality and medical human rights, gender-competent doctors with adequate knowledge and understanding of gender and sexual orientation can, in the core spirit of holistic medicine, care for patients as if they were family members. Thus, creating an LGBT+ friendly healthcare environment demonstrates a commitment to equality and social justice. An LGBT+ friendly healthcare environment must start with medical education reform. As Verdonk, Mans, and Lagro-Janssen [2] indicated, gender equality must be incorporated into policies and education. Medical professionals must understand the relationship between gender and health, and the ideas concerning gender must be addressed through gender courses in medical education. The promotion and implementation of gender equality and LGBT+ friendly healthcare can be achieved only incrementally through education, thereby necessitating the reform of courses.

In addition, because of rapid changes in society and living environments, various educational and learning method reforms have been proposed worldwide. Newly emerged competency-based medical education (CBME) demands the innovation of education and curricula [3,4,5]. Competency has become a prominent education concept in the field. CBME emphasizes the integration of knowledge, attitudes, and skills and the application of these to real life and medical practices [4,5,6,7]. Integrating gender education with CBME requires more research and practical teaching experience. Moreover, the key component of successful education reform is truly the determination of how to inspire medical students to willingly participate in gender-related education and courses.

In particular, we face the following challenges present in contemporary educational environments: (1) Learning has become complex because of diversified learning environments, spaces, and resources, and (2) learning must be immediate, interactive, and fun. In such an environment, medical education professionals must establish effective teaching methods with which to inspire students to learn about gender and to nurture their gender competency. Yang [8] indicated that students who grew up in the digital era only responded to learning methods that were delivered in real time, required participation, and were fun. This requires game-based learning. Students do not want to be inculcated with certain ideologies or values, but rather prefer to be consulted, entertained, and inspired.

Overall, the expertise of teachers in competency-based education and gender education is critical for medical students to understand the importance of gender equality, to enhance gender competency, and to promote the health and well-being of the LGBT+ community in particular.

### 1.1. Competency-Based Medical Education and Gender Competency

Competency refers to the knowledge, attitudes, and skills that learners acquire from education and that enable the handling of complex personal or social scenarios, demands, or endeavors [9,10,11,12]. Mulder et al. [10] proposed characteristics of competency according to an integral perspective and indicated the following points: (1) Competency refers to the collective integration of knowledge, skills, and attitudes; (2) when exhibited in certain professional fields, organizations, tasks, roles, scenarios, and endeavors, competency enables a person to effectively solve problems; (3) competency is embedded in certain situations, and its meaning and accomplishment criteria are given by the specific task context; and (4) competency is demonstrated through behaviors or task orientations.

To effectively care for the health needs of the general public, education reform on the basis of CBME emerged. CBME emphasizes a learner-centered teaching method and entails changing education methods with respect to course development, instructional design, and frequent and formative evaluations that focus on the application of knowledge to practicing skills [3,9]. Teaching and evaluations in CBME focus on the development of integrated abilities in students. The establishment of evaluation and feedback mechanisms during the process of learning teaches students to apply their competencies to real medical scenarios. CBME endeavors to transform knowledge-centered learning methods in conventional school education into integral learning that focuses on cultivating students’ knowledge, attitudes, and problem-solving abilities to accommodate future changes in society and medical practices [3,6].

In 2004, Taiwan implemented the Gender Equity Education Act, which clearly established that gender equity education must include sexuality education, gay and lesbian education, and relationship education. Taiwanese scholars and practitioners of gender equity education include gay and lesbian education in their use of the terms gender education and gender competency. In other words, gender equity education is a general term and includes gay and lesbian education. Therefore, the connotations of the term “gender competency” used in this study include basic gender-related concepts and education regarding sexual orientation and gender identify.

To integrate gender into medical education, Yang and Yen [13] adopted the theory of CBME and gender competency to develop medical education and gender competency indicators (MEGCIs) as a tool for curriculum guidance and instructional design. The MEGCIs framework was divided into three educational phases containing 8 domains, 20 themes, and 79 competence indicators. The domains included “sex and gender”, “gender, health, and medicine”, “diagnosis and treatment”, and “psychiatry and gender.” The themes included “gender and society”, “sexual and gender minorities”, “gender-friendly medical care”, “equality, differences, and power”, and “common sex and gender issues in clinical services” [13]. Yang and Yen argued that the gender competency of medical students comes from the integration of learning regarding gender knowledge, attitude, and skills and the development of abilities relating to appropriate actions. Yang and Yen replaced skills with actions to highlight the connotations of skills and to emphasize that the goal of gender equality education is for students to apply their learned skills to practice and actions.

Many scholars also require medical education to instill correct knowledge of and positive attitudes toward LGBT+ into medical students or healthcare professionals [14,15,16,17,18,19,20]. Many studies revealed the importance of teaching medical students to understand basic LGBT+ concepts and terminology and focused on guiding students toward establishing friendly attitudes toward LBGT+ in their courses [18,19], including inviting LGBT+ community members to participate in curriculum development, lesson planning, or teaching assistance [18]. Some studies demonstrated multiple approaches to providing students with teaching on LGBT+ health in medical education curricula, such as didactic lectures, student-led presentations, patient panels, and small-group sessions [17,20].

Keuroghlian, Ard, and Makadon proposed sexual health education and provided LGBT affirming healthcare environments to advance LGBT health equity. They addressed the mastering of basic LGBT concepts and terminology and the demonstration of openness toward LGBT people as core components of LGBT health education in clinical training programs [19]. Salkind, Gishen, Drage, Kavanagh, and Potts introduced a compulsory curriculum in a medical school to provide undergraduate students with LGBT+ health-related education, including talks from transgender patients as guest speakers. The respective research results showed that students learned to use appropriate language to explain and discuss sexual orientation and gender identity [18].

This study confirms that the teaching of basic LGBT+ concepts and terminology is the first step toward developing medical students and healthcare professionals’ LGBT+ competency and LGBT+ friendly healthcare. This study also asserts that in the process of learning basic LGBT+ concepts, students also learn positive attitudes and skills relevant to LGBT+ healthcare in medical curricula. It adopts the doctrines of CBME and the reasoning that neither curricula and teaching nor teaching and learning are separate in education; competency-based gender curricula and teaching involve a teaching implementation process in which curriculum design and teaching plans are considered simultaneously.

### 1.2. Game-Based Teaching

Game-based teaching and learning is a participatory educational approach in which students brainstorm together to solve problems. Studies indicated that teaching activities involving games stimulate active learning and enhance learning motivation and outcomes [5,21,22,23,24].

Gee [25,26] stated that a successful game-based teaching approach features the following characteristics: (1) Identification, wherein participants establish a sense of identification in the game; (2) interaction; (3) risk-taking, that is, compared with real life, failing in a game does not incur serious consequences, thereby giving participants the freedom to take risks; (4) autonomy, that is participants have control over the game; (5) well-ordered problems, that is, the game is properly designed to include problems that are related and that enable participants to gradually grow and develop; (6) challenging, whereby the game is designed to have problems that challenge the existing professional knowledge of students; (7) instant feedback, whereby students instantaneously obtain necessary information to improve their ability of critical thinking; (8) situated and meaningful learning, that is, students can learn new concepts through game scenarios; (9) pleasantly frustrating; (10) exploration, in-depth understanding, and rethinking, whereby the game forces players to expand contextual knowledge to conduct comprehensive and in-depth thinking; (11) opportunities and environment for team-work; and (12) problem-based learning, where game-based teaching is also a type of problem-based learning which develops the problem-solving abilities of students. The instant feedback from the game helps students to improve and apply their problem-solving skills.

The present study contends that teaching and learning are two sides of the same coin, as learning involves the interaction between learners and teaching content, including teaching activities and media, and this interaction determines what the students learn or experience. Therefore, adopting appropriate teaching methods is crucial.

In summary, in today’s educational environment, medical education professionals must develop an appropriate teaching method to facilitate the development of student gender competency. If CBME is an education approach involving ability-integration learning and if game-based teaching is a powerful method for active learning, then the integration of these methods to teach CBME gender courses could yield novel insights based on the disclosure and review of practical knowledge at the teaching site.

Using the CBME perspective and gender courses in psychiatric clinical education, this study explores whether the application of game-based teaching activities promotes gender learning and improves the gender competency of students. Moreover, on this basis, suggestions are offered regarding competency-based teaching methods. The results of this study could enable teachers to understand which teaching methods and strategies nurture the gender competency of medical students and may help teachers to develop professional skills and competency-based teaching through which to further promote LBGT-friendly healthcare environments.

## 2. Materials and Methods

This study adopted a qualitative case-study methodology, with Kaohsiung Medical University (KMU) as the research setting. KMU is a long-established medical university with affiliated medical centers and institutions. Taiwan has three gender institutes across the country, one of which is KMU. KMU has also committed itself to promoting gender mainstreaming in higher education, including reforms in gender and medical education. The characteristics of case studies are that the cases themselves are empirically or theoretically representative. By analyzing situations, events, or exceptions of general significance, a case study can grasp and present insights at the grand or macro level [27]. Favorable case studies have universal and general requirements and have the effect of echoing and improving theory or practice.

### 2.1. Teaching Design and Implementation

Taiwan’s clinical education is a part of undergraduate medical education and is taught in the fifth or sixth year of college. Medical students in the fifth or sixth year are both students and interns. KMU arranges for a fifth-year or sixth-year medical student to go to psychiatry for one month of clinical education and practice in psychiatry.

Psychiatric clinical education training includes six courses on clinical topics in psychiatry. The six course topics and content include: (1) Communication skills, including mental examination, suicide, and violence assessment; (2) psychopharmacology; (3) cognitive dysfunction, including dementia, delirium, and Mini-Mental State Examination testing; (4) anxiety disorder; (5) substance abuse; and (6) depression. These six courses are taught by different teachers, each for one hour at a time. With the consent of the administrative department of clinical education in psychiatry, the research course, LGBT+ Health and Medical Care (LGBT+ HMC), was added to the psychiatric clinical education training for one academic year. During this year, students entering psychiatric clinical education training were required to take seven courses. Of these seven courses, LGBT+ HMC was the only two-hour course.

This study adopted the CBME perspective and MEGCIs [13] to design the LGBT+ HMC, with an aim of cultivating and enhancing gender competency in medical students through game-based teaching. The teaching objectives of LGBT+ HMC were as follows: (1) To understand how social structures, culture, and other relevant influences affect the lives of LGBT+ individuals, and (2) to perceive the influences of sexual orientation, gender expression, and sexual discrimination on the physical and mental health of LGBT+ patients.

LGBT+ HMC content included basic concepts of gender, such as sexual orientation, LGBT+ issues, sexual and gender identity, the history and social medical background of homosexuality being removed from DSM-5, and related psychopathological theories. The LGBT+ HMC also included content based on the aforementioned teaching goals, such as discussions around how stigma affects LGBT+ mental health, medical assistance to promote LGBT+ health, and more. After the course content was planned, effective design and transformation was still required to promote student learning effectiveness. Therefore, the typical teaching procedure of the course was as follows: Warm up, concept explanation (e.g., what is LGBT+), storytelling (LGBT+ individuals were invited to share their life and healthcare experiences), teaching activities, and course conclusion. This course was conducted by two teachers in rotation, and both teachers were also psychiatrists and attending doctors.

According to the competency-based course and teaching design ideas, a 3 × 3-grid game was designed as the warm-up game for LGBT+ HMC. Apart from stimulating learning motivation, this game served as an evaluative tool, functioning as a pretest and a formative evaluation. The aim of this game was to prompt learning motivation. It was designed to assess students’ prior LGBT+ knowledge, which provided feedback that served to inform the adjustment of the course and the improvement of learning conditions.

The 3 × 3-grid game contained nine questions. Based on the concepts in CBME, these questions were designed to elicit responses regarding students’ understanding of LGBT+ healthcare and mental health issues and could be answered from the perspectives of knowledge, attitudes, and skills. This game was played in teams. The teacher first divided students into two teams that would compete against each other. After the starting order was decided (using the game “rock–paper–scissors”), one member from each team took a turn selecting and answering questions. After an item number was selected, the teacher revealed the question by clicking on the number. The student was required to answer the question within a limited amount of time. The team that gave the correct answer could continue selecting questions, and the team that connected a line of correct answers won. In some instances, the game was adapted into an individual competition with the same rules and procedures. In addition, teachers could ask students to elaborate on certain answers or to answer other relevant questions, introducing real LGBT+ healthcare scenarios and prompting students to consider whether different responsive measures were required for different scenarios and cases. The teacher could also consider the answers from the students to discern their knowledge, attitudes, and skills regarding LGBT+ medical and healthcare.

### 2.2. Participants, Data Collection, and Data Analysis

A total of 230 students entered psychiatric clinical education training and were taught in stages during one academic year. The number of students per stage was 8–12. The LGBT+ HMC was conducted from September 2017 to May 2018, and two teachers were responsible for teaching the course.

Because of concerns regarding research ethics, such as the disclosure of students’ grades, the participants were invited to be interviewed only after the end of the academic year. Invitations to participate in this study were issued only after May 2018. However, some students received invitations to interview almost one year after the end of the course, at which time some had left the school, were undertaking internships at other hospitals, or were preparing for exams and unable to participate in this study. Finally, 19 medical students and two teachers participated in this study (Table 1). In addition to being medical teachers and psychiatrists, the two instructors were also lecturers on gender education courses, often giving speeches on LGBT+ related topics and medical education.

Through semi-structured, in-depth interviews, this study interviewed 19 medical students and two teachers from LGBT+ HMC to obtain feedback and reflections regarding learning and teaching for this topic. Teachers and students were interviewed individually. For each interviewee, one or two interviews were conducted; each interview lasted between 1 and 2 hours. The interviewers used voice recordings and took notes during the interview process, allowing participants to talk freely. The interview data were transcribed by research assistants and translated verbatim.

The researcher participated in each course and took down field notes. The field notes were used to check the interviewees’ feedback on teaching and learning for the course. Field notes were also used as a reference for data analysis. In addition, although few students participated in this study, saturation of information was achieved from qualitative interviews when similar content was repeatedly collected and new information was no longer obtained from respondents.

Data analysis used the perspectives of CBME, gender competency, and game-teaching methods. Thematic analysis was used to analyze the data and themes were identified from the interview data.

### 2.3. Ethical Considerations

To enable students to comment freely on the course, the researcher invited students to participate in interviews only after the course completely ended. Therefore, some students received interview invitations almost one year after the end of the course, some received invitations six months later, and some received invitations one month later. However, at the time when they participated in this research interview, students had completed clinical education and internships at KMU. In addition, all student interviews were conducted by research assistants. The students knew that the researcher was a KMU teacher, that the course was designed by the researcher, and that the researcher participated in each class. Considering these factors, as well as the possibility that students’ responses or opinions were influenced by the need to give socially desirable answers, student participants were interviewed by research assistants. The first interview between the two teachers was also conducted by research assistants. Based on the results of the first interview, the researcher conducted a second interview with the teachers to complete the data collection.

With respect to the disclosure of informed consent, the researchers informed the participants of the place and manner in which the interview would be conducted, that the interview would be recorded and a transcript produced, and that the participants could withdraw from the research at any time. To ensure the research ethics and anonymity, all of the cited interview data were presented as codes. In addition, some quotes were moderately edited, without alteration of the original meaning, for readability.

## 3. Results

### 3.1. Games Gave Students a Sense of Participation and Benefited Knowledge Transmission and Transformation

This study determined that the 3 × 3-grid game successfully provoked learning motivation by arousing curiosity and drawing attention to LGBT+ healthcare issues.

Playing the 3 × 3-grid game at the beginning of the course drew our attention. We could roughly understand what this course was about.

(Student C)

With the 3 × 3-grid game, we were more concentrated in class because the game had our attention at the beginning. Normally in a block course we are always playing with our phones. So yes, I think that game was quite important…Because the way this game proceeded was with competition. We like competitive games; these games draw our attention.

(Student A)

The slides following the game explain the questions in the 3 × 3-grid game…I remember the teacher talked about the process of removing homosexuality from being listed as a mental disease…Although I know that homosexuality is not a disease, I did not know this revolution process and historical development, etc. When the teacher explained the process, I was like, oh…oh…, it was like this! One of the questions mentioned that homosexual and bisexual individuals experience higher [rates of] mental troubles compared with heterosexual individuals. I found this information quite informative…I think these questions should be considered for people to assess why are they like that.

(Student D)

The learning behaviors and learning outcomes of students were easily affected by the teaching method [28]. Students repeatedly mentioned that “playing games caught our attention from the beginning,” that games “drew our attention,” and that “the 3 × 3-grid game made us concentrate.” The research thus determined that game-based teaching effectively reduced students’ resistance to learning about LGBT+ subjects.

The students stated that, in courses conveying knowledge in conventional lecture form, they often played with their phones. Moreover, the competitiveness and participation involved in games attracted students to participate in learning and motivate them to acquire previously unknown knowledge. As already described, students who grew up in the digital era only react to learning methods that are delivered in real time, are interactive, and are fun [8]; game-based learning satisfies these criteria. These students desire guidance, entertainment, and inspiration. The 3 × 3-grid game, which enabled students to participate, interact, challenge, and take risks, represents such a teaching and learning context.

In addition, using this game as a teaching activity aimed to achieve two objectives: To trigger the learning motivation of students and encourage them to proceed to concept learning, and to prevent lecturing on LGBT+ concepts from becoming an instance of the “banking education” [29]. Student responses also revealed that game-based teaching helped teachers convey and integrate knowledge or concepts into a game; students connected with the course content and experienced knowledge transformation to achieve positive learning outcomes [30]. Game-based teaching not only helped the transmission of knowledge but also provoked thinking and exploration for students. Students were able to learn about LGBT+ related topics in a game that expanded their existing cognition, attitudes, and skills.

With respect to LGBT+ medical and healthcare issues, within the context of a game, students were able to express and discuss their correct, incorrect, or even biased understandings regarding LGBT+ communities without being overly concerned with providing politically correct answers. These interactions and dialogues led to meaningful learning. Moreover, students expressed their desire to conduct further dialogue and learning.

There was one question where the teacher just gave out the answer. I think the teacher should let us discuss it before telling us the answer or the teacher’s point of view; we can listen to why some people think it is bad. However, doing so may have one disadvantage, which is the lack of time; also the discussion might lead to some intense arguments, hehe, just like that…I think the time reserved for the 3 × 3-grid game was too short. I wish the discussion time could be longer…The teacher should not talk all the time and appear to be superior…I wish for more discussion time because I would like to hear from people holding different opinions; I would like to know why they insist on their own point of view regarding LGBT+ communities.

(Student X)

Weinert [12] indicated that competency was only formed through learning processes rather than through direct inculcation. This study revealed that not only LGBT+ concepts, but also attitudes such as respect for the healthcare rights of LGBT+ individuals, awareness of sexism, and respect for gender equality, must be taught through enlightenment. Moreover, the mere transmission of knowledge without encouraging the learning process provoked no transformation in attitudes, because such teaching methods constituted mere preaching or inculcation of students with unquestioned ideologies.

This study revealed that competency requires a learning process. Teaching and learning were situated in an intensely and dynamically uncertain interactive scenario, and teachers were required to respond to dynamic tensions and challenges according to students’ performance. This topic is discussed in the following section.

### 3.2. Games Embodied the Idea of Assessment as Learning; the Pleasant Frustration from the Game Motivated Students to Learn

The most critical step in a competency-based curriculum project is the identification of students’ abilities [9]. Therefore, the 3 × 3-grid game constituted a teaching activity as well as a learning task. The concepts of assessment as learning (AaL) were employed, and the assessment was designed to be part of the game. The purpose of AaL is to help teachers obtain feedback on their teaching on the basis of adjusting their teaching methods to help students learn [31]. Therefore, AaL emphasizes the integration of assessment into the process of teaching and learning and transforms the assessment into part of the teaching activity and learning process.

In fact, students were clearly aware of the assessment purpose of the 3 × 3 grid game after taking this course.

Actually, the 3 × 3-grid game is kind of like a pretest to test everyone’s understanding of this issue.

(Student A)

I prefer the 3 × 3-grid game…because it is unlike other classes. Most of the other classes only involve the teacher explaining slides to us and this kind of interaction is rare…Lectures are for us to absorb information, or rather, teachers throw us information without us having to think about it. The game involves more interactions and gives us a chance to weigh our knowing with LBGT medical care.

(Student W)

Assessments can be performed in numerous forms, including exams, pencil and paper tests, or presentations. Block curricula, which medical departments often employ, usually adopt exams as the formative or conclusive evaluation. The response from students indicated that the integration of competitive games into the interactive process of teaching and learning and games as an assessment tool was popular. Games provided an indication of learning objectives to students. As stated by one student: “We could roughly understand what this course was about.” This statement revealed that AaL is a two-way street: Teachers could determine the abilities of students, and students could also evaluate their own understanding of course topics.

The game served as a warm-up activity to stimulate learning motivation and to enhance students’ concentration. It also enabled teachers to obtain instant feedback. The assessment was therefore helpful for both teaching and learning, engendering positive interactions and a reflection circle. The game serving as AaL was helpful for the teacher to determine students’ abilities, prior knowledge, and relevant understandings regarding LGBT+ healthcare.

For example, when the researcher and the teachers were designing the questions for the 3 × 3-grid game, the answers to some of the questions were considered basic knowledge that students must know and be capable of understanding. Surprisingly, none of these students, who had already commenced their internships in a hospital, could state the year in which homosexuality was removed from the classification of mental diseases. Numerous students did not know what “transgender” meant, and a few students were unfamiliar with the meaning of LGBT+.

I think the 3 × 3-grid game was quite fun, compared with lectures. On one hand, the game made us think and was interactive…Because in the medical department, regular classes are carried out by the teacher lecturing without knowing whether the students are listening. However, I think starting a course with this game was very intriguing. For example, one of the questions asked in which year homosexuality was removed from the classification of mental diseases and we really did not know that…Then, you become curious about what the other questions are…I feel like it drew my attention. I think it was quite interesting.

(Student D)

As previously stated, successful game-based learning is adequately challenging and pleasantly frustrating [26]. In games, students are frustrated by not being able to answer a question. Experiencing challenges is also positively stimulating in a game and provokes motivation to learn among students; for example, one student stated that “You become curious about what the other questions are, and that draws my attention.” The students were more focused and invested in the following section of the course, which explained the concepts of LGBT+ health issues and psychiatry. The game achieved the exploratory function of game-based learning and provoked active thinking in students.

Learning outcomes refer to quality experiences/knowledge/practices that students accumulate through meaningful learning in a situation; they emphasize the true abilities of students rather than scores or grades [32]. This study revealed that the 3 × 3-grid game, which served as the teaching activity and assessment tool, also supported learning and teaching. The game helped with learning by identifying difficulties and misconceptions experienced by students, which enabled the timely provision of instructional scaffolding to enhance efficacy. For example, the course was designed to conduct concept teaching after the game, and the concepts included the history of the removal of homosexuality from the classification of mental diseases (including the modification of DSM-5) and an introduction to LGBT+ communities. The teachers also invited LGBT+ individuals to the class to share their own medical experiences. These real-life experiences gave the answers to the questions in the game and helped the students with the integration of knowledge, attitudes, and skills.

In addition, the game facilitated teaching because the design of the game allowed for the integration of assessment into teaching, which made the assessment part of the teaching method and teaching activity rather than a supplementary evaluative tool. In fact, both aspects of the game enhanced assessment for teaching and exhibited value in terms of instant feedback obtained in the CBME and game-based learning.

### 3.3. Discussion is the Key to Deepen Competency Learning and Improve Teaching and Learning Effectiveness

I felt like we had more time to think this time, like the 3 × 3-grid game at the beginning gave us some questions to think about. Before, classes were infinite lectures without giving us any time to think about why we think like this or act like this…Now that we could truly think about LGBT+ issues through discussions, learning will not be just listening to what the teacher has to say and forgetting about it immediately.

(Student B)

Conventional learning models require students to learn first instead of undertaking any practical activities and testing their abilities. By contrast, in game-based teaching, learning and skills are acquired actively through games [25,26].

I think the 3 × 3-grid game can be mixed with group discussions. The teacher should hold on to the answer and even withdraw from nodding or showing any emotions. The teacher should simply ask: “Why do you think like this?” So, everyone knows we can express our opinions. Otherwise, if the person answering the question happens to know the answer, the teacher will not be able to listen to opinions or answers from other people. So, the teacher can obtain feedback that there are other ideas. Besides, I like and want to have open discussions. Some questions have no correct answers. Sometimes, even if there is a correct answer, you can still listen to the other opinions or thoughts. In certain moments, these other opinions or thoughts are maybe what we really need to pay attention to.

(Student K)

Student feedback revealed that the process of teaching is extremely complex and unpredictable. Any planned teaching activities could be altered at the teacher’s discretion, and decisions regarding follow-up teaching plans or actions were influenced by the engagement levels of students during implementation at the education site. Student feedback also revealed that they wanted further discussion of LGBT+ healthcare issues after the game. As such, the teachers were asked how they thought this desire for discussion was inspired by the game.

Time management is essential. Whenever I had to sacrifice something in class, it was usually the discussion part.

(Teacher A)

Arranging such interactive activities are great, but I think it is not easy. It is not easy because course design, choosing teaching materials, and designing an activity take a lot of time. The preparation alone demands the investment of a huge amount of time. Then, the process of teaching also consumes time and energy. Giving a lecture or simply talking is easy. However, activities involve a lot of uncertainty at the site, such as lack of response from students or a great variety of responses. The teacher must be able to handle the responses and respond to them cleverly. Whenever I failed to handle a response or give a proper response, I would feel so frustrated afterwards. Therefore, having an activity is more energy-consuming than simply giving a lecture.

(Teacher B)

According to Rovegno [33], determining the teaching knowledge of teachers requires an understanding of events in the classroom. Because CBME focuses on the learner and the process of learning, teaching activities must be modified accordingly [6]. The feedback from the teachers and students revealed that a well-designed teaching activity, such as the 3 × 3-grid game, could indeed inspire students to think and provoke student participation and teacher–student interactions, potentially prompting interrelated questions and more discussions. The student feedback revealed that the discussions offered room for reflection in competency-based learning and improved the effectiveness of game-based learning. However, teachers may not always be available to conduct discussion promptly in the classroom.

Reasons for unavailability to conduct discussions included a lack of confidence by teachers with respect to conducting discussions or the anxiety of being unable to handle situations following discussions. The 3 × 3-grid game was a teaching activity and a teaching method. Whether to conduct discussions or increase discussion time depends on the scope and depth of a teacher’s teaching knowledge, such as being familiar with numerous methods by which to conduct a discussion or being able to lead and respond during discussions.

CBME emphasizes the integral learning of knowledge, attitudes, and skills. The present study revealed that, for gender competency learning, although these three aspects can be separated, they coexist and interact. This was explained in student feedback: “Sometimes, even if there is an answer, you can still listen to other opinions. At certain moments, these other opinions may be what we really need to pay attention to.” This statement revealed that discussions not only clarify education on aspects of LGBT+ healthcare, but also change attitudes toward LGBT+ communication and LGBT+ friendly healthcare provision.

Discussions are a teaching method involving cognition, attitudes, and skills. Moreover, leading discussions is a learning process that transforms attitudes and changes opinions. One aspect of competency-based teaching is for students to participate in or conduct discussions [34]. Therefore, teachers must focus on discussions in competency-based teaching and use discussion and dialogue as a basis for all education, reflection, and action. Integrating games into gender competency courses and teaching requires follow-up discussions to clarify the gender competency abilities of students during the interactive aspects of the game.

## 4. Discussion

In 2004, Taiwan implemented the Gender Equity Education Act, which clearly established that gender equity education must include sexuality education, gay and lesbian education, and relationship education. Gender equity education began to encounter huge obstacles in 2014 (i.e., 10 years after the Gender Equity Education Act was created and came into force). Numerous religious groups and parents used “traditional family values” as an excuse to oppose gay and lesbian education. They claimed that only two genders exist, men and women, and that gay and lesbian education could transform children into gays or lesbians. In Taiwan, people who disapproved of gays or lesbians severely opposed gay or lesbian allies. In 2018, Taiwan held a referendum. The voting results constituted an overwhelming victory for opponents of gay and lesbian education. In addition, a referendum proposition that gender equity education be enhanced failed to pass, accompanying the requests to revise the Gender Equity Education Act and to prohibit the use of the words “gay and lesbian education”. Extremely varied understandings of and attitudes toward the LGBT+ communities exist in Taiwan.

The question is whether students are mature enough to understand LGBT+ issues. Students’ feedback cited in the article indicated that they did not fully understand LGBT+ issues. Using students’ responses on page 8 as an example, this study found that some medical students did not even understand what the acronym LGBT+ represents or what the term transgender means. The course in question was provided for clinical education in psychiatry. The researcher participated in observing the course for an entire academic year. When questioned regarding which year homosexuality ceased to be considered a disease, almost no students could answer. According to Student D, students who participated in this study indicated that they did not understand the history of how homosexuality ceased to be considered a disease, and they had no knowledge in this area. By interviewing students, it was observed that numerous medical students had prejudices against LGBT+ people or exhibit hostile attitudes toward these communities.

It is for this reason that the purpose of this study was to emphasize the importance of education first. Medical doctors have a high level of social prestige and status in Taiwanese society and are highly influential. This study aimed to explore effective courses and teaching designs and methods to integrate gender equity education into medical education, thereby cultivating students’ gender competency and achieving the ultimate purpose of LGBT+ friendly medical care.

Undoubtedly, games motivate students to learn. A well-designed game, especially one that corresponds to the learning characteristics of complements students’ learning styles such as competitiveness, achieves the learning objective regarding the integration of multiple abilities in CBME. The 3 × 3-grid game used competitions and contests to guide students’ thinking, exploration, and development of relevant concepts and enhanced students’ participation and active learning. In addition, the game inspired students’ desire to learn and opened up opportunities and spaces for discussion between teachers and students.

Nevertheless, using games as a teaching method and a teaching activity requires relevant knowledge of teaching design. A factor that limits opportunities for discussion is a lack of time. In fact, this problem is related to course planning and decision-making in classes, and these aspects are part of teaching design. Each teaching activity is designed to help students achieve certain learning objectives and learning outcomes. However, these objectives and students’ accomplishments do not always align in reality, which is a challenge of competency-based teaching. The 3 × 3-grid game featured the AaL attribute. Therefore, in addition to providing positive feedback and encouragement to students regarding their responses in class, teachers were required to pay attention to ideas, misconceptions, or values that the students presented and provide further guidance in terms of thinking and conducting discussions.

In game-based teaching, teachers play a crucial role. Teaching has been conceptualized as a design science [35], where teachers are designers of learning [25]. Gee emphasized that games are a useful tool, but the way in which positive learning outcomes are facilitated using such tools and methods must be determined [22,25]. In fact, all teaching design models generally include the five steps of analysis, design, development, execution, and evaluation. Thinking, exploration, interactions, and discussions all require time for implementation, organization, and reflection. Adequate time to learn and assimilate are indispensable learning moments, even when a game is fast-paced, and must be incorporated into the teaching design, planning, and execution.

Gee indicated that students must experience all types of situations to benefit from learning activities [22]. A well-designed game provides numerous educational experiences. In particular, a successful learning activity requires participation in social groups. Games offer the experience of sharing and interaction with others through discussions, conversations, interactions, and modeling between peers and teachers. With specific experiences, learning brings in-depth understandings and improved problem-solving abilities. A well-designed game-based learning activity provokes reflection as well as the reconsideration and reconstruction of specific values, and benefits the learning of cognition, attitudes, and skills. This was demonstrated, for example, in the above-mentioned quote from a student who expressed a desire for further exploration of the ideas relating to the questions they had already answered.

Overall, a successful game-based teaching approach must be designed by the teacher and integrated into appropriate courses for students to receive meaningful learning outcomes. In this manner, playing a game can be an effective learning approach rather than just a form of entertainment. In particular, teachers must analyze, design, develop, and evaluate students’ learning performances and allocate adequate learning time for them to experience all learning moments to optimize the effectiveness of CBME and game-based teaching.

A limitation of this study was that, although 19 student participants allowed for “saturation,” the quality of the research results could have been enhanced by using more participating students to achieve “sufficiency”. Also, whether the study’s voluntary student participants exhibited a degree of selection bias was unable to be evaluated; for example, whether these participating students had friendlier attitudes toward LGBT+ communities or were interested in LGBT+ related subjects. As discussed previously, teaching is a methodology [35]. Effective teaching effectively transfers course content to students through effective teaching methods and the design of teaching activities to achieve predetermined learning results. If a teaching method fails to attract the attention of and motivate students that are interested in a topic, the teaching method and strategy are deemed invalid.

LGBT+ education is a highly controversial education issue in Taiwan. Students who were willing to participate in this study half a year or one year after the end of their courses, while also graduating or preparing for national examinations, were potentially more likely than most people to care about LGBT+ related subjects and LGBT+ healthcare. If game-based teaching promoting LGBT+-friendly healthcare gained the academic interest of these students and motivated them to participate in this study, their opinions are likely meaningful and valuable. This reinforces the spirit and benefit of case studies.

Comparatively, the small number of students participating may have reflected the shortage of students concerned regarding LGBT+ friendly healthcare. This was the purpose of the CBME education reform. In response to criticism that medical education overlooked social responsibility in the past, CBME emphasizes social accountability and effective care for the health needs of the general public [3,4]. The results of this study indicated that game-based teaching and CBME could be used to effectively provide students with teaching regarding gender and LGBT+ competency. Students interested in LGBT+ issues can act as seeds. For young people, the influence of peer education sometimes exceeds that of formal education and curricula. If students’ attitudes regarding LGBT+ issues can be understood in the process of data collection, a more complete reference might be available for analysis and interpretation of this study, thereby preventing bias.

Finally, the teacher’s feedback indicated that, although game-based teaching is an effective method to engage students in gender and medical competency education courses, investing in such teaching methods poses quite a challenge for teachers. Teachers’ professional development must rely on institutional support systems and the resources provided by schools.

## 5. Conclusions

In the current education environment, effective gender competency learning for medical students requires strategic teaching methods that integrate gender into courses and create integral learning of knowledge, attitudes, and skills through interactive teaching activities. The present study revealed that designing gender competency courses by incorporating games enhanced the concentration and interest of students. Moreover, the instant assessment and feedback enabled students to understand their achievements and directions in learning. The challenge and pleasant frustration experienced during the game were stimulating and encouraged learning. At the same time, students were willing to actively think and explore, thereby taking initiative in the learning process and providing a multifaceted learning and reflection process that established cognition, attitudes, and skills. In this competency-based course and teaching approach, the game served as the teaching method, teaching activity, and assessment tool.

Moreover, this study revealed that game-based teaching was helpful to convey and integrate knowledge. One objective of the gender education course was the elimination of bias and discrimination against LGBT+ communities and in LGBT+ healthcare. Game-based teaching activities and learning tasks familiarized students with LGBT+ related knowledge and concepts. Also, these knowledge and concepts could be immediately transformed into attitudes and skills.

However, this study also revealed that applying game-based teaching in a competency-based curriculum challenged the teacher’s ability to design courses and steer discussions. In the course of teaching, where the environment changes but the learning content remains, teachers must be flexible enough to handle the essentials of teaching design and improve their knowledge and practical abilities regarding discussion-leading. This ability is a core proficiency for teachers in the practice of CBME. The teacher’s ability to lead a discussion is a key factor to deepen and affect the gender competency learning of students. If a teacher cannot truly master the challenges of leading classroom discussions and facilitating the acquisition of practical knowledge and experiences, the teacher’s professional knowledge on education remains suspended in the meaningless realm of abstraction.

This study revealed that, for teachers to improve their ability to lead a discussion and design a teaching activity, they must strengthen their pedagogical knowledge of theories and professional proficiency; more critically, they must reflect on, react to, and exploit their teaching practice experiences, thereby modifying their teaching strategies and transforming their teaching knowledge to improve teaching effectiveness and achieve change.

For gender and medical education professionals, social changes refer to the goals of eliminating sexism and ushering in a gender-equal society through relevant courses and classroom teaching. In light of the trends and developments in CBME, the results of this study provide a reference for teachers on the basis of how to learn or improve their abilities to teach gender competency so that the spirit of equality can be encouraged in medical students.

This study addressed the application of games in regard to the development of teaching activities, the design of learning assessments, the reform of teaching methods, the improvement of teacher knowledge, and the enhancement of pedagogical proficiency. The actual improvements in gender competency exhibited by medical students who participated in this research demonstrate the design of gender competency CBME courses with game-based teaching. Moreover, this study provides a reference for medical teachers and professionals seeking a novel means by which to integrate gender competency into clinical education and improve teaching knowledge.

## Figures and Tables

**Table 1:** The basic sociodemographic data of the participants.

Participants	Sex		Years of Study		Age Range	In total
Students	Male	Female	Fifth year	Sixth year	23–30	19
	11	8	11	8	19	
Teachers	2	0				2
